# Improvement in Suction Plates

**Published:** 1854-07

**Authors:** Charles H. Dubs

**Affiliations:** Dental Surgeon.


### ARTICLE IX.

*Improvement in Suction Plates.* By CHARLES H. DUBS, Dental Surgeon.

THE value of this improvement consists in effecting an equal or superior force of atmospheric pressure and adhesion of the metal, and with or without the central cavity. It leaves the palatal organ entirely unrestrained and uncovered by the metallic plate, burdens the mouth with less weight of metal, and depends for its power of adhesion by atmospheric pressure entirely upon the deepening of the rugæ, particularly those in or near the crown of the dental arch.

To effect this, in the first place, I take two exact impressions of the mouth, and if there is any choice between them, reserve the best impression for the second cast, or dies.

Secondly, I make my model in sand, cast the dies, stamp the plates well, then take the reserved wax impression, and carefully cut out and deepen the lines of all the rugæ or undulations that are visible, in depth about three times the thickness of a sheet of foolscap paper. I then run my plaster as usual, and before the plate is completely struck up, I planish out the palatine edge of the plate and proceed to stamp; but, previously, I cut a thin piece of copper plate, as thick as a half dime, and in width one-eighth of an inch, formed to follow the entire palatine edge of the plate, stamp it up in the first dies, trim to suit exactly the edge of the plate and then stamp as usual, retaining the copper plate between the lower palatine surface of the metallic plate and the antagonizing dies, and when the plate is complete I set the teeth and finish.

In cutting deeper all concave parts of the plate, or the lines of rugæ or undulations of the mouth, I am very particular to preserve the natural form and relative proportions of the same. A plate made on this principle does not require a width of more than half or five-eighths of an inch from the back part of the lining of the incisor teeth to the palatine edge of the plate.

If a central cavity is desired, I take a piece of copper or annealed brass plate, the thickness of a dime, cut it in an oval crescent form, one inch in length by half an inch in width, more or less, according to the size of the mouth. Placing the same between the two first dies, I stamp its form. At the time of its being struck up, the piece of copper or brass plate should be larger than needed, and afterwards trimmed to the suitable size with the edges square. With this mode of obtaining the central cavity, it is very neat when finished, and corresponds exactly to the deepened rugæ of the mouth. To get the depression for the central cavity, after being struck up, I cast on my last die, by placing the copper or brass plate on the male cast, which has received a faint impression, although sufficient to hold it in the exact position required. Then placing my zinc male model ready to receive the female die, I hold the cavity plate down steadily with a slender piece of iron wire, which can be withdrawn before cooling; I then stamp the plate over, for the last time, with the cavity plate and also with the narrow copper plate that has already been used to keep the palatine edge of the plate for the mouth tight and flat to its approximate surface. When the central cavity is desired, the plate should be three-eighths of an inch, or more, wider than when no other force than the deepening of the rugæ is required. Both of these plans I have used in my dental practice, the one for nine and the other for six years, and I must express it as my candid opinion that those plates depending for adhesion alone upon the deepened rugæ are as good, or better, for the majority of mouths as those with the central cavities. In the former, every deepened rugæ has the power of the cavity, prevents the plate, while in the mouth, from being forced down, and brings the bearing and pressure of the plate to a firm rest upon the concave part of the arch of the mouth, capable, by its structure of bone and cartillage, thus leaving the soft, pulpy and fleshy part of the convex of the mouth, entirely at ease, free from constraint, pressure and irritation.

I can truly say, that in my practice, I never, on this principle, had a case attended with the least irritation, but all have given entire satisfaction.

*Natchez, July 17th,* 1854.

